# Parametric Study of the Reflective Periodic Grating for In-Plane Displacement Measurement Using Optical Fibers

**DOI:** 10.3390/s120404265

**Published:** 2012-03-28

**Authors:** Yeon-Gwan Lee, Dae-Hyun Kim, Chun-Gon Kim

**Affiliations:** 1 Department of Aerospace Engineering, KAIST, 335 Gwahangno, Yuseong-gu, Daejeon 305-701, Korea; E-Mail: yeongwan@kaist.ac.kr; 2 Department of Mechanical and Automotive Engineering, Seoul National University of Science and Technology, 172 Gongneung 2-dong, Nowon-gu, Seoul 139-743, Korea; E-Mail: dkim@snut.ac.kr

**Keywords:** optical fiber, sinusoidal signal, one-way displacement, grating pattern

## Abstract

This paper presents a technique for a simple sensing principle that can be used for the measurement of displacement. The proposed sensor head is composed of a reflective grating panel and an optical fiber as a transceiver. The simplified layout contributes to resolving the issues of space restraints during installation and complex cabling problems in transmission fiber optic sensors. In order to verify the effectiveness of the proposed technique, it is important to obtain the sinusoidal signal reflected from the grating for reasonable phase tracking. In the numerical analysis, a real wave based optical beam model is proposed for the extraction of predicted signal according to the grating width and ratio of reflection bar width to spacing. The grating pattern design to obtain a sine wave reflected sensor signal was determined within an R-square value of 0.98 after sine curve fitting analysis. Consequently, the proposed sensor principle achieved the in-plane displacement measurement with a maximum accuracy error of 5.34 μm.

## 1. Introduction

Fiber optic sensors are promising candidates for application in real-time structural health monitoring (SHM) techniques [1,2] due to their immunity to electromagnetic interference. Over the past few years, many types of optical fiber sensors such as the extrinsic Fabry-Perot interferometric (EFPI) sensor [3,4] and the fiber Bragg grating (FBG) sensor [5] have been developed, and these types of fiber optic sensors offer significant advantages. In particular, the FBG sensor has been widely adopted in the commercial industrial field due to its multiplexing [6]. However, while most intensity based sensors are relatively low cost in the development of sensor systems, FBG sensors are expensive in order to set up wavelength shift interrogation systems. Among the intensity modulation techniques, reflective fiber optic sensors (FOSs) [7–9], including interferometer FOSs [10], have been researched using concave and flat mirrors. However, most reflective FOSs have been limited to applications in very small measurement ranges of a few millimeters to microns [11]. Accordingly, in order to develop economical and widely applicable optical fiber sensors for extended measurement ranges, studies have been conducted on hydrophone [12] and accelerometers [13–15] that use the transmitted light of FOSs through two grating panels [12–15] or a grating film [16] to directly measure the dynamic displacement of a seismic mass. Essentially, these techniques function using the sinusoidal signal of transmitted light that is patterned based on the shutter or moiré fringe effect of two grating panels, and they have satisfied the basic requirement of low cost in developing a sensor system. However, fabricated prototypes of such sensors have demonstrated disadvantages due to their complex design, which features four optical fiber lines on both sides: each side has two optical fiber lines for a light emitter and receiver. Therefore, this leads to limitations in installation of the fiber optic accelerometers (FOAs) due to the required geometric conditions. Furthermore, the problem of complex cabling is intensified when the FOAs are used to monitor large size structures through multipoint sensing in hazardous locations.

In this study, in order to change the complex structure of the sensor to a simpler one, a new technique is proposed to obtain the sinusoidal signals using a single reflective grating panel and two optical fibers as transceivers. The proposed sensor is designed as follows: the emitted light from one optical fiber arrives on the grating panel and then the partially reflected light from the grating enters the optical fiber that is used as a light emitter. As a result, the main distinctions between the proposed design and other accelerometers [12–16] that are also based on the grating panel are the use of a single reflective grating panel (RGP) to vary the intensity of the reflected light and the use of only two transceiver optical fibers on one side of the sensor case. However, in order to operate as a conventional grating based sensor, it is critical that a stable and periodic sine wave signal be obtained from the reflected light that is induced by the movement of the grating panel. If the sinusoidal signals are acquired as a sensor signal based on the new principle, the directional information of the displacement can be obtained using two optical fiber lines of which the signals are in quadrature, as do most sinusoidal output sensor types such as the quadrature phase shifted (Q) EFPI [17] and the moiré fringe optical fiber sensor [13,14]. Therefore, this paper is limited to the consideration of a parametric study of the grating pattern to obtain only a sinusoidal signal using one optical fiber.

In order to confirm the proposed design, numerical analyses were carried out to investigate the characteristics of the reflected signal and the restricted conditions for sinusoidal signal acquisition, and experiments were also performed to verify the reflected signals according to the grating pattern. Through comparisons of the simulation and experimental results, the reliability of the simulation model technique was verified. Furthermore, the grating pattern design required in order to obtain a stable and periodic sine wave as the output signal was determined. Therefore, this technique realizes the advantages of the reflection grating sensor, which has a simpler and more effective design than conventional transmission grating sensors.

## 2. Sensor Principles

**Figure 1.**
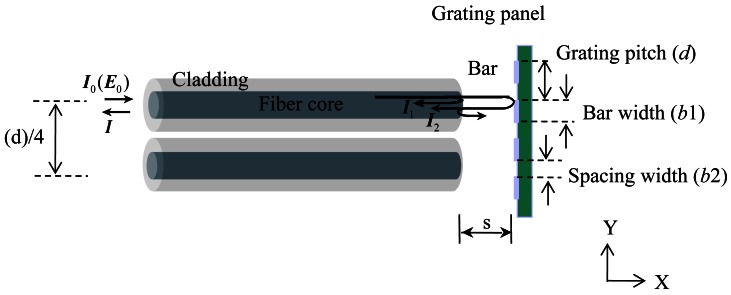
Schematic diagram of the signal acquisition.

This section explains the main sensing mechanism, which measures the displacement of a grating using optical fibers. The two optical fibers with a separation of quarter grating pitch are depicted in Figure 1 in order to comprehensively explain the sensor principles although only one optical fiber was used in this paper for reflected signal verification. An RGP consists of multiple bars with evenly spaced spacing; the bar width (*b*1) and spacing width (*b*2) are smaller than the optical beam width. The length *s* refers to the air gap between the end of the optical fiber and the RGP. *s* is fixed during the sensor operation. Initially, light is emitted into the air at the outlet of the optical fiber core. Then, the emitted light is reflected on the bars, which serve as mirrors. The reflective light intensity is constant in the initial state, as shown in Figure 1: *I*_0_ and *E*_0_ refer to the light intensity and electric field of the input laser, respectively.

However, if the RGP moves in a perpendicular direction to the optical fiber resulting from the excited force, the total reflective light intensities *I* can be expressed as follows:

(1) $$I=I_{1}+I_{2}+I_{12}$$

where *I*_1_ refers to the reflective light according to Fresnel's law, *I*_2_ refers to the reflective light on the grating area, and *I*_12_ refers to the interference term between *I*_1_ and *I*_2_.

Although *I*_1_ caused by the Fresnel's reflection at the glass-air interface is constant, *I*_2_ and *I*_12_ vary with the vertical movement of the grating. If *d* is constant and equal to the sum of *b*1 and *b*2, then when the RGP shifts by one *d* in the *Y* direction, the reflected signals to the two optical fibers will also shift by one *d*. Moreover, when the optical fiber is fixed as an observer and the RGP is attached to an observation measurand, the quantification of the measurand displacement is accomplished based on the periodic signal of the reflected light. As a result, if the phase of the periodic signal can be tracked, the displacement of the measurand can be measured due to the value of *d*, which is equal to the period of the sinusoidal signal. This is the basic sensing mechanism of the grating panel optical system. In particular, when the periodic signal is sinusoidal, the signal processing for the phase tracking will be more reasonable and logical with this system. Consequently, the raw reflected signals *S_1_* and *S_2_* can be expressed as sinusoidal functions with a phase difference of 90°. After normalization to a sinusoidal function, which has an amplitude of one and a mean magnitude of zero, the two normalized reflected signals $\bar{S_{1}}$ and $\bar{S_{2}}$ were expressed as follows with *d* and displacement *u*(*t*) [13,14]:

(2) $$\bar{S_{1}}=\cos(\frac{2\pi \cdot u(t)}{d})$$

and:

(3) $$\bar{S_{2}}=\sin(\frac{2\pi \cdot u(t)}{d})$$

*u*(*t*) can be inferred by unwrapping the two signals as follows:

(4) $$u(t)=\frac{d}{2\pi }\cdot \text{unwrap}(\tan^{-1}(\frac{\bar{S_{2}}}{\bar{S_{1}}}))$$

Theoretically, the beam profile should have a Gaussian distribution. If the light, which has a Gaussian intensity profile, is reflected from the periodic grating panel and the panel moves with a constant speed, the intensity of *I* is sinusoidal in restricted conditions. In order to have a sinusoidal intensity *I*, it is critical to determine the restricted conditions of the grating pattern in which a stable and periodic sine wave signal is obtained. Therefore, a parametric study of the reflective grating pattern should be considered according to *b*1, *b*2, and the grating ratio *m* of *b*1 to *b*2 in order to obtain a sinusoidal signal using one optical fiber.

## 3. Numerical Analysis

In order to describe the signal reflected on the grating, the reflected signals that depend on *b*1, *b*2, and the ratio of *b*1 to *b*2 were simulated as the movement of the grating normal to the optical fiber.

### 3.1. Assumptions and Optical Beam Model

A gap loss, which deforms the reflected wave form [18], is not important parameter. It is only related to light collecting efficiency. However, before developing the simulation, the loss amount, which is induced by the air gap *s*, is analyzed theoretically in order to obtain a more logical reflected light magnitude with regard to light collecting efficiency [19–22]. In this paper, the normalized reflectance neglecting interference term is of interest for a fixed *s*. Smaller separation *s* is ideally desirable because the light collecting efficiency increases with a decrease in the separation between the OFs and the reflective mirror. Accordingly, the loss amount was calculated when *s* was assumed to be 0.01 mm. Six factors, which were used in calculation of the loss amount, are described in Table 1.

The coefficients of the reflectance (*r*_e_) and transmittance (*t*_e_) at the end surface of the optical fiber were assumed to be 0.180 and 0.820, respectively. The coefficients of the reflectance at the surface of RGP (*r*_g_: 0.500), radius of optical beam (*R_beam_*: 61 μm), numerical aperture of fiber (*NA_fiber_*: 0.225), and laser wavelength (*λ_laser_*: 1.550 μm) were as assumed in the simulation. The calculated light loss was approximately 18% of the incident light. Therefore, the assumptions of the numerical analysis were as follows. First, the light intensity at the outlet of the optical fiber is 1; next, there is perfect transmission on the spacing areas and reflection on the bar areas, and only parallel light is emitted to the RGP; finally, the 18% loss of the total reflected light was caused by the distance of 0.01 mm.

The cross section of the optical fiber core is a circle, and it has different light intensities at each location. For these reasons, when the RGP moves, each point of the bars reflects a different light intensity. Therefore, it is clear that the light intensity distribution is a critical factor in the simulation because the form of the reflected signal depends heavily on the light intensity distribution. Consequently, the light intensity distribution should be considered in the simulation.

**Figure 2.**
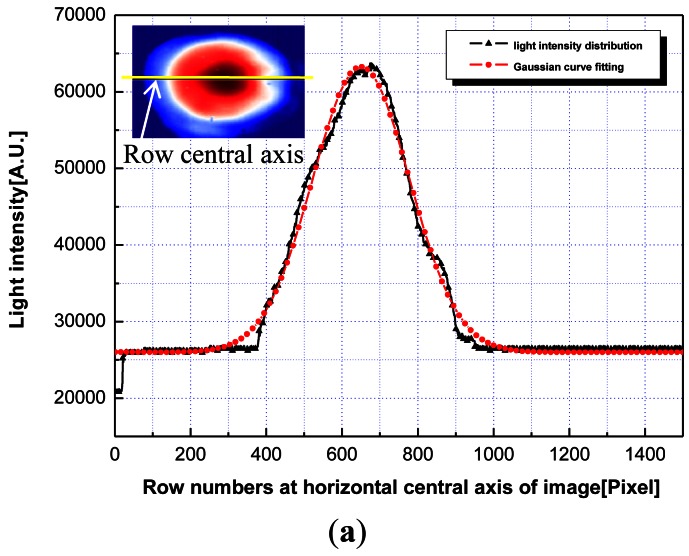

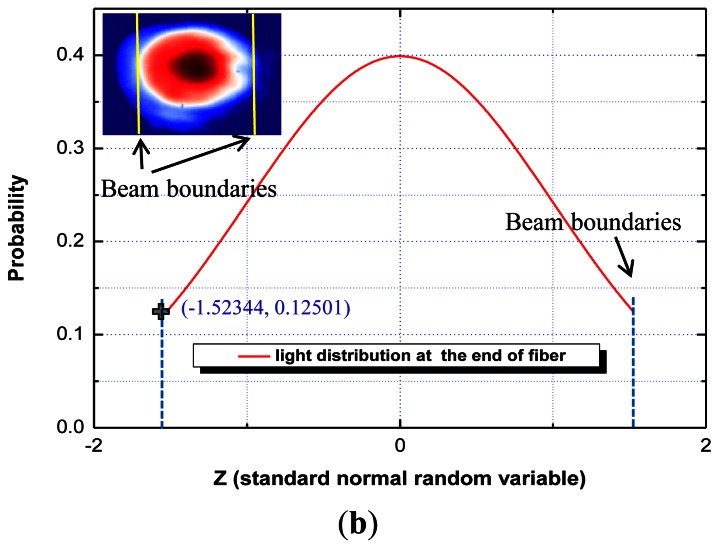
Beam profile test results. (**a**) Gaussian curve fitting analysis; (**b**) Standard normal distribution.

For a real wave based model, the beam profile information is a prerequisite for describing the reflected optical signal. Thus, a beam profile test was undertaken using a multi mode fiber (MMF) combined with a collimator (LPC-05-1300-M, Oz Optics Co., Ottawa, Canada) and a 1,550 nm laser diode based on the charge-coupled device technique. Figure 2(a) shows a near field pattern image and a beam profile of the row central axis at the smoothed core end face of an optical fiber. The beam profile had a slightly elliptical shape, and the form could be assumed to have a Gaussian distribution. Based on the results of the Gaussian curve fitting analysis, the R-square values of 0.990 and 0.986 were obtained for the horizontal central axis and perpendicular central axis of the image, respectively. Also, for the data in the boundary lines in Figure 2(b), the Gaussian approximation formula could be transformed into a standard normal distribution, which can be expressed as follows [23]:

(5) $$f(Z)=\frac{1}{\sqrt{2\pi }}\cdot e^{-\frac{z^{2}}{2}}\;,\text{where}Z=\frac{X-650}{128}$$

**Figure 3.**
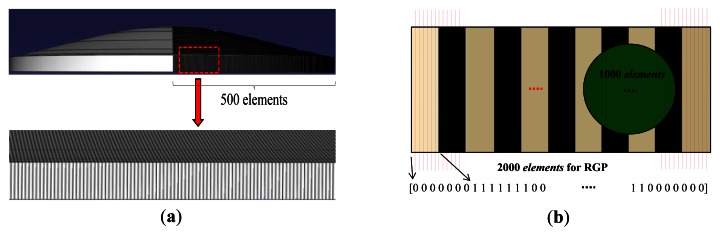
Schematic models for simulation. (**a**) Meshing using commercial software, ABAQUS 6.7; (**b**) Model of the RGP contains multibar and spacing.

The obtained standard normal distribution equation was employed to draw the external form of the spatial light intensity distribution using ABAQUS 6.7, as shown in Figure 3(a). The model was divided into 1,000 meshes. After the volume of each mesh was divided by the total volume, the probabilities of the light intensities in each mesh were obtained; then, this information was fed into optical beam model of the simulation code.

In Figure 3(b), the optical beam (array of *A*) and RGP (array of *B*) were modeled with 1,000 and 2,000 *elements*, respectively, using MATLAB 7.1. The obtained spatial light intensity probability of each mesh was assigned to the optical beam model in turn. For the RGP, the *elements* in the bar area and spacing area were assigned as 1 and 0, respectively. Only parallel light is emitted to the RGP and only parallel light can be reflected. With this assumption, when the optical beam model is projected to the RGP model, each assigned 1 or 0 values of the RGP *elements* were multiplied by the scalar spatial light intensity probability value, which is assigned on the optical beam *element* opposite the RGP. In terms of the optical beam model, 1,000 *elements* of the optical beam model (array of *A*) are multiplied to each face to face value of 1,000 *elements* on the RGP model (array of *B*). Then, all the multiplied values were summed as follows:

(6) $$\text{Reflection}(n)=\sum_{i=1}^{1000}\left(A_{i}\cdot B_{(n-1)+i}\right)\;\text{where}\;n=1\;\text{to}\;2000.$$

**Figure 4.**
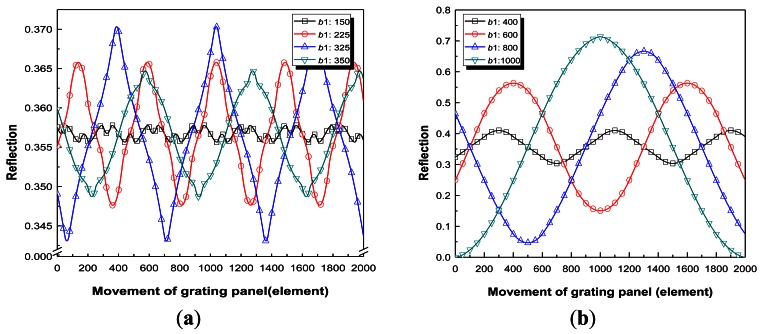
Variations in the reflective light depending on *b*1 when *m* = 1 (case 1). (**a**) Less than 400 *elements* (*b*1); (**b**) More than 400 *elements* (*b*1).

**Figure 5.**
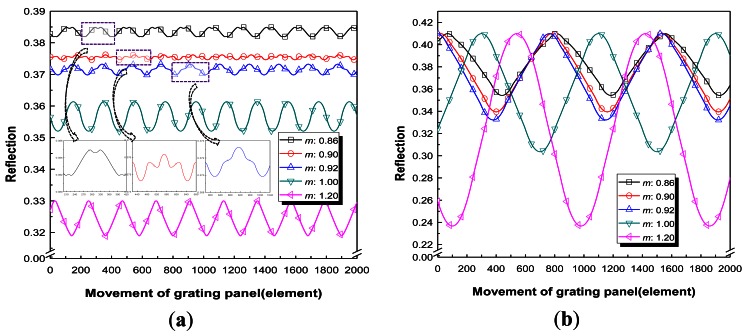
Variations of the reflective light according to different grating ratios (case 2). (**a**) Variations of the light depending on *m* when *b*1 = 100 *elements*; (**b**) Variations of the light depending on *m* when *b*1 = 400 *elements*.

The summation value indicates the reflected optical intensity at one position. In order to describe the modulated optical intensity as the RGP moves, the *element* arrays of the RGP model are shifted by one *element*. Therefore, the sum of *A_i_*·*B_(n−1)_*_+_*_i_*, where *i* equals 1 to 1,000, were repeatedly calculated at every position and plotted by iterations from *n* equals 1 to 2,000. Consequently, as the bar width is equal to the beam width, reflected light with at least over one period can be simulated because RGP were modeled with two times (2,000 *elements*) of optical beam *elements* (1,000 *elements*). Therefore, this simulation describes the variation of reflected light intensity using iterations of the grating element changes based on real measured beam profile information. Figures 4 and 5 describe the reflection function of *n* (movement of grating panel).

The simulation was then undertaken to determine the characteristics of the reflected signal and the grating pattern design for sinusoidal signal acquisition.

### 3.2. Simulation Results

The bar width *b*1 and *b*2 are defined as an *element* number according to a relative proportion to the whole number (1,000 *elements*) of the optical beam element. They can be expressed as percentages with regard to the whole element number of the optical beam model. Therefore, the optical beam size is a criterion for conversion to the physical dimensional unit in the experiment. If the incident optical beam size was measured as a physical dimensional unit such as micron or millimeter, the *element* size can be converted to a physical dimensional unit by dividing the incident physical optical beam size by 1,000 *elements*. In this way, in order to determine the grating pattern to be required for the sinusoidal signal acquisition, the reflected signals were investigated based on the real wave based model according to *b*1, *b*2, and grating ratio *m*, which was defined as follows:

(7) $$m=\frac{b2}{b1}$$

They were divided into two cases. In case 1, the effects of variations in *b*1 on the reflection were investigated when *m* was 1. The number of *elements* inputted for *b*2 was set equal to *b*1 for all cases. In case 2, the effects of variations of *m* were studied when *b*1 was fixed. Figures 4 and 5 show the results for each case; these Figures are plots of the values of reflection on one fixed optical fiber *versus* the movement of the RGP in the normal direction to the parallel bars of the grating, as shown in Figure 1. The horizontal axis of Figures 4 and 5, which plots the reflection intensity according to *n* as the iteration number, represents the movement of RGP according to the single *element* shifts, as described by Equation (6). The modulated reflected signals during the movement equal to twice the whole element number (1,000 *elements*) of the optical beam model were obtained because the RGP was modeled with 2,000 *elements*.

**Figure 6.**
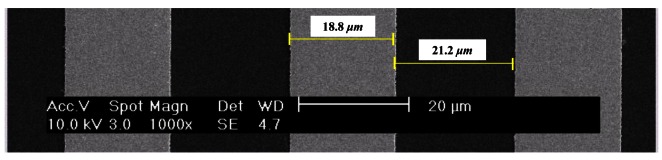
SEM images of surface at 20.0 μm grating.

Variations of the reflective light are plotted in Figure 4(a) with bar widths lower than 400 *elements*. Although the reflected lights are almost sinusoidal signals except special cases (bar widths of 150, 225, 325, and 350 *elements*), which are described in order to demonstrate the unusual signals when the bar widths were below 40% of the beam width. Furthermore, the variations of the reflective lights are plotted in Figure 4(b) when the bar widths were beyond 400 *elements*. All the cases (both below and beyond 400 *elements*) investigated in the reflected signal simulation had bar widths with 1 *element* increment starting from the bar width of 1 *element*, where the spacing widths were set equal to the bar widths within each case. The variations of the light depending on the grating ratios at bar widths of 100 *elements* and 400 *elements* are plotted in Figure 5(a,b), respectively. Furthermore, the results from Figures 5 and 6 are summarized in Table 2. The R-square values after sine curve fitting analysis were analyzed through commercial OriginLab 8.0 (OriginLab, Co., Northampton, MA, USA).

From the results of case 1, when the bar widths are below 40% of the optical beam width the R-square values are low. Although the case with a *b*1 of 350 (*elements*) has an R-square value of 0.9961, the form of the reflected light is not a sinusoidal signal. Unlike a sine function, a gradual slope suddenly changed into a steep slope. When this grating pattern is realized in the intensity modulator of the sensor, reflected light in this form produces a large error for tracking the displacement of the moving RGP because it is difficult to express one sine function as in Equation (2). It was concluded that when the bar widths are below 40*%* of the optical beam width as the RGP moves, various forms of nonlinear periodic signals in some RGPs were obtained as a result of the nonlinear summed combinations of reflected light at each location. However, when the bar widths exceed 40% of the optical beam width as the RGP moves, the shapes of the reflective signals are almost periodic sine waves within an R-square value of 0.996. Furthermore, the results of the analyzed periods are in good agreement with the theoretical periods within a 1% margin of error. The magnitude was also analyzed because this value directly represents the signal to noise ratio (SNR). The wider the bar is, the larger the peak-valley amplitude is. This implies that the SNR can be increased with increased *b*1 when *m* equals 1.

From the results of case 2, it was concluded that when the bar widths are below 40% of the optical beam width, the shapes of the reflective signal varied for each *m* as the RGP moves, as shown in the inset of the Figure 5(a). The R-square values were 0.9666 (*m* = 0.86), 0.5748 (*m* = 0.90), and 0.9435 (*m* = 0.92) at a *b*1 of 100 *elements* based on the sine curve fitting analysis, respectively. These three ondulatory reflected signals cause the signal processing to be impossible for the measurement of displacement because it cannot be expressed mathematically as a sine function for reasonable phase tracking. However, when the bar widths exceed 40*%* of the optical beam width, then as the RGP moves, the shapes of the reflective signal were almost periodic sine waves within an R-square error of 2% based on the sine curve fitting analysis. This means that in order to obtain a sinusoidal reflected signal regardless of the small changes in *m*, the bar widths should exceed 40*%* of the optical beam width. The results of the analyzed periods are in good agreement with the theoretical periods within a 1% margin of error. Consequently, it is desirable that the bar widths exceed 40% of the instant beam width, and when this condition is satisfied, variation of *m* from −0.2 to +0.2 can be allowed for sinusoidal reflected signal acquisition.

## 4. Experiments

### 4.1. Fabrication of Reflective Grating Panels

In order to verify the simulation results, a total of four RGPs with individual bar widths of 20.0 μm, 40.0 μm, 62.5 μm, and 90.0 μm were designed for the mask. Gold was plated on the quartz wafer as the mirror and wet etching was used to remove the gold plating in order to obtain spacing areas.

Figure 6 shows the SEM images of the fabricated RGP. *b*1 was smaller than *b*2 in the fabricated RGP due to the anisotropic etching rate used to produce the spacing areas. In the case of the RGP of 20.0 μm, *b*1 is 18.8 μm (± 0.13 μm) and *b*2 is 21.2 μm (± 0.13 μm) under the assumption that *d* was 40.0 μm. In a strict sense, the fabricated RGP has an *m* of 1.13 (± 0.015). However, for convenience, the dimension of the bar in the mask design is representative of each RGP.

### 4.2. Signal Verification System

In the experiment, a broadband source (BBS1550 + 2FP00, JDS Uniphase) was used as a light source and a five-axis jig with a micrometer lever was also used in order to adjust the alignment of the optical fiber and the RGP. The distance between the end of the optical fiber and the RGP was maintained at 0.2 mm in order to protect the RGP from damage induced by contact and friction.

**Figure 7.**
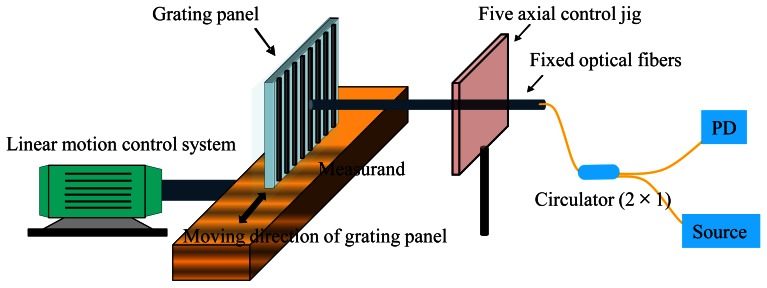
Configuration of the experimental setup.

The separation distance of 0.2 mm was measured and guaranteed using the micrometer of the five-axis jig. Then, the perpendicular-alignment state between the OF and RGP was maintained using the five-axis jig during the signal verification experiment. The RGP was moved using a continuous motor system, as shown in Figure 7. The reflective light was obtained through the circulator (PICT-1550-S, Oyokoden Lab, Tokyo, Japan) and photo detector (ν2011, New Focus, Inc., Santa Clara, CA, USA). Each of the 10,000 bits of data on the reflective light, which was received using the multi mode fiber (MMF) combined with the collimator, was stored in a data acquisition system at a sampling rate of 2.5 kHz.

### 4.3. Experimental Results

**Figure 8.**
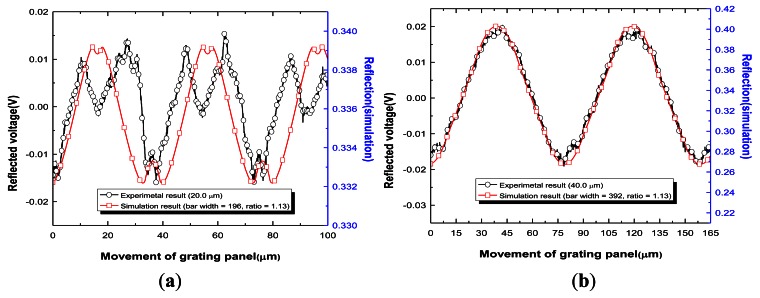

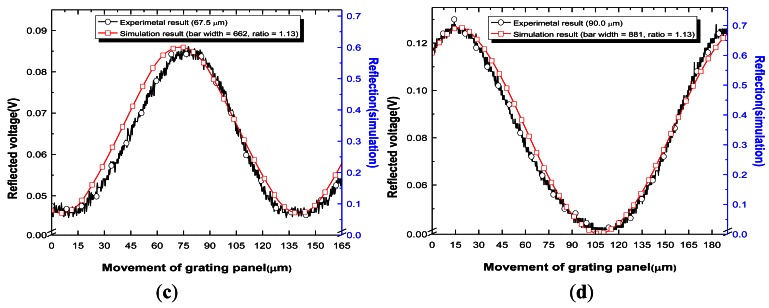
Measurement of reflection signals according to grating pattern. (**a**) RGP with *b*1 of 20.0 μm; (**b**) RGP with *b*1 of 40.0 μm; (**c**) RGP with *b*1 of 67.5 μm; (**d**) RGP with *b*1 of 90.0 μm.

Before the design of the RGP was implemented, a beam width test was performed. The experimentally measured Gauss beam widths were 93.5 μm and 97.0 μm at the central row and column axis, respectively. The optical beam width was assumed to be 96.0 μm because the optical beam was of elliptic form. *d* is constant and equal to the sum of *b*1 and *b*2 of the mask design. In the simulation, the size of 1,000 *elements* was set equal to 96.0 μm. Therefore, one element represents a physical dimension of 0.096 μm. In addition, *m* of 1.13 was applied to the simulation. For example, in the RGP with 20.0 μm, 196 *elements* (±1.4 *elements*) in the simulation indicates 18.8 μm (±0.13 μm) of *b*1 and 221 *elements* (±1.4 *elements*) indicates 21.2 μm (±0.13 μm) of *b*2, which is identical to the dimensions of the fabricated grating described earlier in Figure 6. Figure 8 shows the experimental results compared with the simulation results for the four RGPs. The horizontal axis indicates the one-way movement of the RGP. This movement was calculated using a linear motor velocity (180 μm/s) with a sampling rate of 2.5 kHz in the reflected light. When the bar widths fell below 40% of the beam width, as shown in Figure 8(a), the output signal shape in the experiment was consistent with that in the simulation and was a nonlinear wave. However, when the bar widths exceeded 40% of the beam width, periodic sine wave signals were obtained, as shown in Figure 8(b,d).

For more detail, the R-square values, periods, and magnitudes of the simulation and experimental results are compared in Table 3 based on a sine curve fitting analysis. The bar widths of 20.0 μm, 40.0 μm, 67.5 μm, and 90.0 μm respectively correspond to 21%, 42%, 70% and 94% of optical beam width (96.0 μm).

The R-square value 0.4394 of the RGP (20.0 μm) in the experiment was lower than the simulation result (0.9573) because the calculated period based on the sine curve fitting analysis in the experiment was 18.5 μm due to a non-sinusoidal periodic form. However, the forms of reflected signals for both were similar. Although an R-square value of 0.9573 in the simulation was not low, both forms cannot be admitted as a reasonable sensor signals. In both forms, the undulatory signals were acquired repeatedly in the form of concave signals at the peak and valley regions. This results in the signal processing to be mathematically impossible for displacement measurement because these reflected signal forms cannot be expressed as a sinusoidal function. From the R-square values as shown in Table 3, for 40.0 μm, 67.5 μm, and 90.0 μm, the form of the reflected signals were fitted with a sine wave within 2% error of the R-square value. In both the simulation and experiment results, the calculated periods from the sine curve fitting analyses were in good agreement with the ideal ones within a maximum error margin of 2%. Furthermore, in the experiments, the one-way displacement of the grating, which is described in the horizontal axis in Figure 8, was indirectly in good agreement with the periods in each RGP. The increasing rate of the magnitude in the simulation according to *b*1 was different when compared with the experimental results. The possible reasons for this discrepancy include the assumption of a loss factor (18%), which was calculated based on the conventional researched uniform wave model [17–20]. However, in both simulation and experiment results, the more *b*1 increased, the larger the amplitude of the reflected signal became. This implies that as the RGP moves, SNR increases with an increase of *b*1. These results correspond well with the simulation results. In the experiment, although the RGPs have an accuracy error regarding *b*1 and *b*2 when the bar widths exceed 40*%* of the instant beam width, an *m* value of 1.13 was allowed for sinusoidal reflected signal acquisition. In terms of the reflected signal forms, periods, and magnitudes, the tendencies of the simulations coincided with the experimental results. Therefore, the reliability of the simulation was verified using the results of the experiments.

### 4.4. In-Plane Displacement Measurement

Based on the experimental setup of Figure 7, one more OF with a separation distance equal to quarter grating pitch from the other OF, was added for in-plane displacement measurement. The RGP with bar width b1 (90 μm) equal to half grating pitch and with grating pitch of 180 μm was used. The grating was attached to the measurand, which was controllable by a linear motion control system. As the measurand moved, the data was measured and saved at sampling rates of 4,000 Hz.

**Figure 9.**
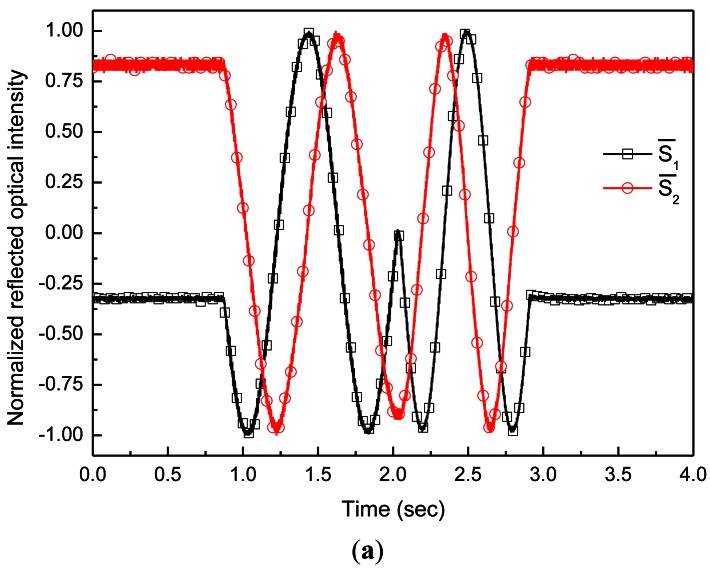

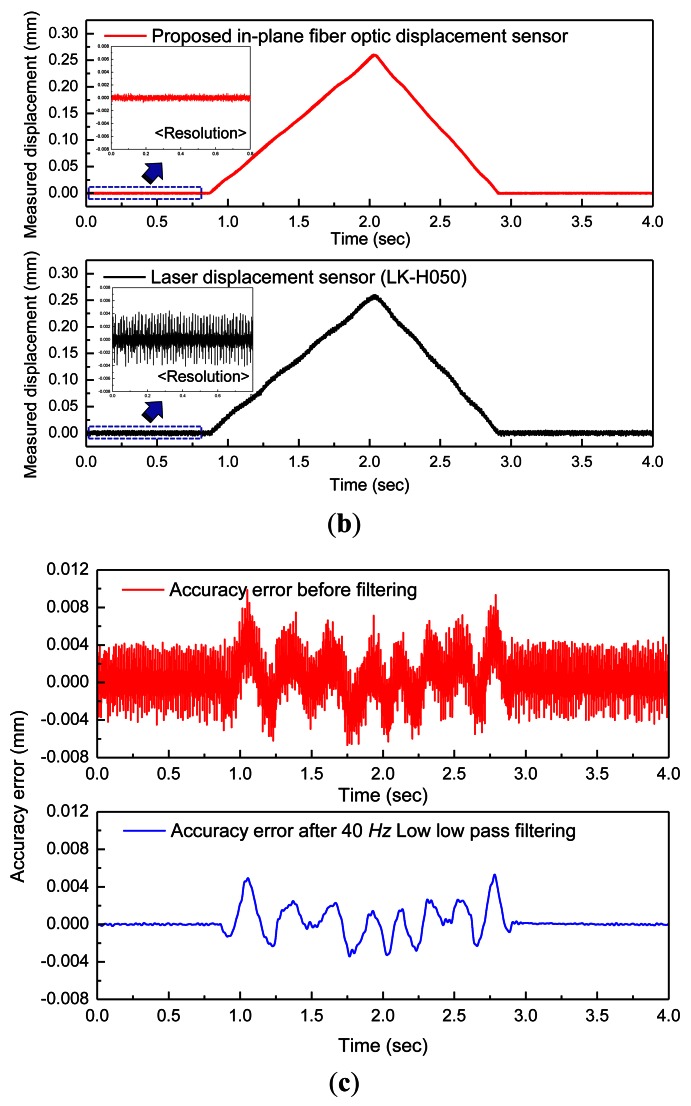
In-plane displacement measurement. (**a**) Normalized reflected signals of the two OFs in quadrature; (**b**) Comparisons of the in-plane displacement measurement by the proposed FOS and LDS (LK-H050); (**c**) Accuracy between the measured displacements by the proposed FOS and commercial LDS.

Figure 9(a) shows the normalized reflected optical signals with 90° phase difference. The two signals can be expressed by the forms of Equations (2) and (3), respectively. Therefore, the relative displacement can be inferred by Equation (3). The measured displacement is described in Figure 9(b) with displacement measured by a commercial laser displacement sensor (LDS; LK-H050, Keyence, Co., Osaka, Japan). In the inset of Figure 9(b), each resolution of the LDS and proposed FOS is described. The peak to peak resolutions of each sensor are 1.5 μm and 8.5 μm at full-bandwidth. The measured displacement of the LDS was significantly affected by electrical noise. Therefore, in order to analyze the accuracy of the proposed FOS properly, the displacement measured by the LDS should undergo low pass filtering in order to eliminate the high frequency noise. After a low pass filtering of 40 Hz was applied, the accuracy between the measured displacements by the proposed FOS and commercial LDS is plotted in Figure 9(c). The maximum accuracy error was 5.34 μm.

## 5. Conclusions

A new working principle for a reflective grating panel-optical fiber sensor has been presented in this paper. The bar widths and spacing widths individually must comprise more than 40% of the optical beam width for a stable and periodic sinusoidal signal acquisition. In terms of reflectivity, when the bar width is equal to the beam width, the signal to noise ratio for the motion of the reflective grating will be maximized. The sensor principle proposed in this paper achieved in-plane displacement measurement with a maximum accuracy error of 5.34 μm. By employing a reflection based technique, the sensor structure was simplified through the use of the optical fibers as transceivers, which allowed the fibers to be placed on only one side of the sensor case. Therefore, the simplified layout contributes to resolving the issues of space restraints during installation and complex cabling problems in transmission fiber optic sensors. Furthermore, after the combination of single degree of freedom system, this measurement concept can be applied to propose novel sensor system for a variety of measurement applications such as tilt angles, accelerations, and so on.

## Figures and Tables

**Table 1.** Coefficients of reflectance (*r*_e_) and transmittance (*t*_e_) at the end surface of the optical fiber, Coefficients of reflectance (*r*_g_) at the surface of RGP, the radius of optical beam (*R_beam_*), the numerical aperture of the fiber (*NA_fiber_*), and the laser wavelength(*λ_laser_*) used in the calculation of the loss amount.

*r_e_*	*r_g_*	*t_e_*	*R_beam_* [*μm*]	*NA_fiber_*	*λ_laser_* [*μm*]
0.180	0.500	0.820	61	0.225	1.550

**Table 2.** R-square values, periods, and magnitudes of each grating pattern via sine curve fitting analysis.

	*m*	*b*1 (*elements*)	R-square value	Period (*elements*)	Magnitude
**Case 1**	1	150	0.6263	299.33	0.002
		225	0.9760	449.86	0.018
		325	0.9630	651.43	0.027
		350	0.9961	700.14	0.016
		400	0.9960	800.66	0.106
		600	0.9998	1200.30	0.413
		800	0.9991	1600.21	0.619
		1,000	0.9999	2006.73	0.713
**Case 2**	0.86		0.9666	185.99	0.003
	0.90		0.5748	190.11	0.002
	0.92	100	0.9453	191.96	0.003
	1.00		0.9961	200.03	0.009
	1.20		0.9926	220.04	0.011
	0.86		0.9892	745.00	0.056
	0.90		0.9924	761.10	0.070
	0.92	400	0.9934	768.96	0.078
	1.00		0.9960	800.66	0.106
	1.20		0.9997	880.62	0.173

**Table 3.** Comparison of the results between the simulation and experiment.

*b*1	Characteristics	Simulation	Experiment
20.0 μm (21%)	R-square value	0.9573	0.4394
	Period (μm)	40.1	18.5
	Magnitude	0.007	36.1 (mV)
40.0 μm (42%)	R-square value	0.9983	0.9940
	Period (μm)	80.1	79.3
	Magnitude	0.135	39.8 (mV)
67.5 μm (70%)	R-square value	0.9996	0.9901
	Period (μm)	131.4	133.7
	Magnitude	0.531	43.0 (mV)
90.0 μm (94%)	R-square value	0.9994	0.9808
	Period (μm)	184.1	176.0
	Magnitude	0.693	98.0 (mV)
